# RSK1 protects P-glycoprotein/ABCB1 against ubiquitin–proteasomal degradation by downregulating the ubiquitin-conjugating enzyme E2 R1

**DOI:** 10.1038/srep36134

**Published:** 2016-10-27

**Authors:** Kazuhiro Katayama, Chiaki Fujiwara, Kohji Noguchi, Yoshikazu Sugimoto

**Affiliations:** 1 Division of Chemotherapy, Faculty of Pharmacy, Keio University, Tokyo, Japan

## Abstract

P-glycoprotein (P-gp) is a critical determinant of multidrug resistance in cancer. We previously reported that MAPK inhibition downregulates P-gp expression and that P-gp undergoes ubiquitin–proteasomal degradation regulated by UBE2R1 and SCF^Fbx15^. Here, we investigated the crosstalk between MAPK inhibition and the ubiquitin–proteasomal degradation of P-gp. Proteasome inhibitors or knockdown of FBXO15 and/or UBE2R1 cancelled MEK inhibitor-induced P-gp downregulation. RSK1 phosphorylated Thr162 on UBE2R1 but did not phosphorylate FBXO15. MEK and RSK inhibitors increased UBE2R1-WT but not UBE2R1-T162D and -T162A expression. UBE2R1-T162D showed higher self-ubiquitination and destabilisation than UBE2R1-WT and -T162A. Unlike UBE2R1-WT and -T162A, UBE2R1-T162D did not induce P-gp ubiquitination. UBE2R1-WT or -T162A downregulated P-gp expression and upregulated rhodamine 123 level and sensitivity to vincristine and doxorubicin. However, UBE2R1-T162D did not confer any change in P-gp expression, rhodamine 123 accumulation and sensitivity to the drugs. These results suggest that RSK1 protects P-gp against ubiquitination by reducing UBE2R1 stability.

The human ATP-binding cassette (ABC) transporter superfamily consists of 48 members^1^,^2^. P-glycoprotein (P-gp)/ABCB1 is one of the ABC transporters and pumps out several anticancer agents from cells, including anthracyclines, *vinca* alkaloids and taxanes, small molecule kinase inhibitors, digoxin, HIV protease inhibitors and statins from cells^2^,^3^,^4^,^5^,^6^,^7^,^8^,^9^. P-gp expression in cancer cells confers the phenotype of multidrug resistance (MDR) to these anticancer agents^10^,^11^,^12^,^13^.

The mitogen-activated protein kinase (MAPK) pathway is one of the most important signalling pathways in cell growth and survival. Receptors of tyrosine kinase, such as epidermal growth factor receptor (EGFR) or vascular endothelial growth factor receptor (VEGFR), are activated by ligand-dependent self-phosphorylation, which subsequently activates the MAPK pathway by phosphorylation of MAPK/ERK kinases (MEKs), extracellular signal-regulated kinases (ERKs) and p90 ribosomal S6 kinases (RSKs)^14^,^15^. The activated ERKs and RSKs translocate from the cytosol to the nucleus and phosphorylate several factors, such as c-Myc, STAT1/3 and C/EBPβ, associated with cell growth, proliferation, differentiation and anti-apoptosis^16^,^17^,^18^,^19^,^20^. In many cancers with an MDR phenotype, this ligand-dependent regulation is deregulated, and the MAPK pathway is consistently activated to obtain powerful cell growth activity.

In our previous studies, MEK inhibitors or siRNAs for *MEK*s, *ERK*s and *RSK*s lowered P-gp expression by promoting its degradation^21^. We also reported that P-gp is degraded by the ubiquitin–proteasome system^22^. P-gp is recognised by SCF^Fbx15^, which consists of S-phase kinase-associated protein 1 (SKP1), Cullin 1 and F-box only protein 15 (FBXO15), and then ubiquitinated by the ubiquitin-conjugating enzyme E2 R1 [UBE2R1, also known as cell division cycle 34 (CDC34) or UBC3]^23^,^24^,^25^. Poly-ubiquitinated P-gp then proteolysed by proteasome. However, the association between the MAPK inhibition-mediated degradation of P-gp and the ubiquitin–proteasomal degradation is still unclear. In this study, we thus investigated the regulation of ubiquitination-related enzymes, FBXO15 and UBE2R1, by the MAPK enzymes and found that RSK1 induced self-ubiquitination of UBE2R1 followed by its proteasomal degradation in a phosphorylation-dependent manner, resulting in the protection of P-gp against degradation.

## Results

### Proteasome inhibitors or FBXO15/UBE2R1 knockdown reduced MEK inhibitor-mediated downregulation of P-gp

**Figure 1:**
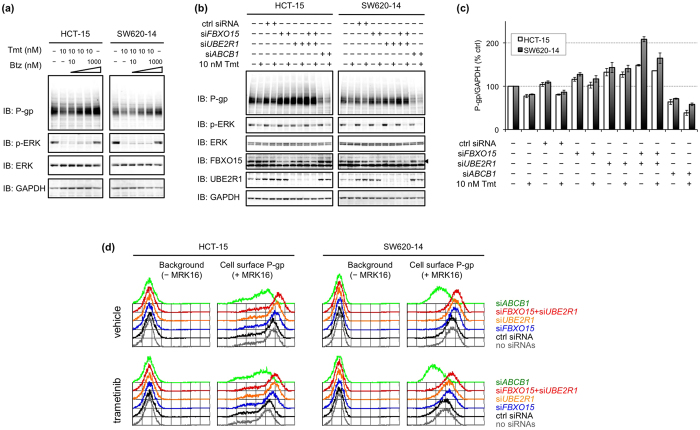
Proteasome inhibitors or FBXO15/UBE2R1 knockdown reduced trametinib-mediated downregulation of P-gp. (**a**) Changes of P-gp expressions in cells treated with trametinib (Tmt) and/or bortezomib (Btz) for 10 h. (**b**) Changes of P-gp expression in cells transfected with si*FBXO15* and/or si*UBE2R1* for 60 h followed by treatment with Tmt for 10 h. (**c**) Graphic representation of P-gp expression normalised by GAPDH expression in (**b**). Each bar represents the mean ± standard error from two independent experiments. (**d**) Changes of P-gp expression on the cell surface membrane. Cells were transfected with si*FBXO15* and/or si*UBE2R1* for 48 h followed by treatment with vehicle or Tmt for 20 h. P-gp expression was determined by flow cytometric analysis with (+MRK16) or without (−MRK16) an antibody to P-gp.

To examine association of the ubiquitination system for P-gp with the MAPK signalling pathway, we firstly investigated the effects of proteasome inhibitors on MEK inhibitor-mediated P-gp downregulation. Human colorectal cancer HCT-15 and SW620-14 cells were treated with MEK inhibitors, trametinib or U0126, combined with increasing concentrations of proteasome inhibitors, bortezomib or MG132. Trametinib lowered P-gp expression, but bortezomib reduced this downregulation in a dose-dependent manner (Fig. 1a). Similar to this, MG132 reduced the U0126-mediated downregulation of P-gp (Supplementary Fig. S1a). Next, cells were transfected with si*FBXO15* and/or si*UBE2R1* for 60 h followed by treatment with trametinib (Fig. 1b,c) or U0126 (Supplementary Fig. S1b,c) for an additional 10 h. Preceding knockdown of FBXO15 or UBE2R1 partially reduced the trametinib- or U0126-mediated downregulation of P-gp, and combined knockdown of both FBXO15 and UBE2R1 further reduced the P-gp downregulation. Flow cytometric analysis was then performed to investigate the expression of P-gp on the cell surface (Fig. 1d). Similar to the results of immunoblotting, cells transfected with either si*FBXO15* or si*UBE2R1* showed partial resistance to trametinib-mediated downregulation of cell surface P-gp, and in cells transfected with both, the downregulation was completely abolished. These results suggest that inhibition of the ubiquitin–proteasome system for P-gp competed against MEK inhibitors-mediated downregulation of P-gp with regard to not only total proteins but also cell surface expression.

### RSK1 and RSK3 bound to UBE2R1

**Figure 2:**
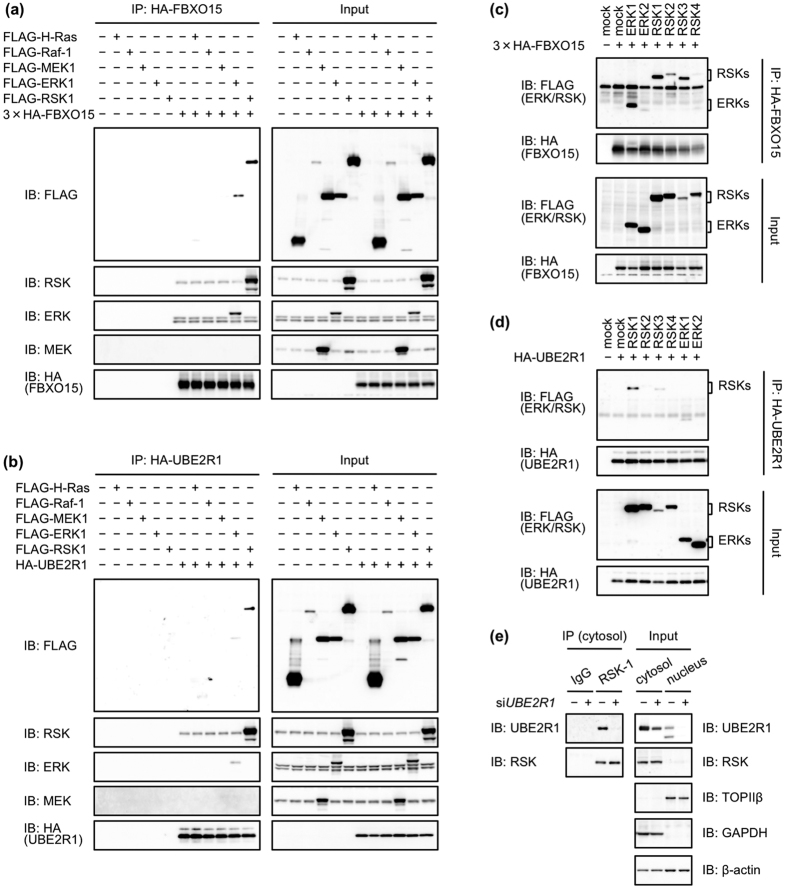
RSK1 and RSK3 bound to UBE2R1. (**a**–**d**) HEK293 cells were transfected with the indicated combinations of plasmids for 24 h. Immunoprecipitation and immunoblotting were performed as described in the Methods section. Binding of ERK1 and RSK1 with FBXO15 (**a**); binding of RSK1 with UBE2R1 (**b**); binding of ERK1 and RSK1/2/3 with FBXO15 (**c**); and binding of RSK1 and RSK3 with UBE2R1 (**d**). (**e**) Endogenous binding of UBE2R1 with RSK1. Cytosolic and nuclear lysates were prepared from HCT-15 cells transfected with non-silencing control siRNA or si*UBE2R1*. Endogenous RSK1 was immunoprecipitated with an anti-RSK1 antibody from the cytosolic lysate. TOPIIβ and GAPDH expression was evaluated to monitor the fractionation into the cytosol and the nucleus.

Immunoprecipiatation-immunoblotting analysis was performed to evaluate the interaction of FBXO15 or UBE2R1 with the enzymes that make up the MAPK signalling pathway. HEK293 cells were cotransfected with 3 × HA-tagged *FBXO15* plasmid and FLAG-tagged *Raf-1*, *MEK1*, *ERK1* or *RSK1* plasmids, followed by immunoprecipitation of FBXO15. As shown in Fig. 2a, immunoblotting with an anti-FLAG antibody revealed that exogenous ERK1 and RSK1 were coprecipitated with FBXO15, but Raf-1 and MEK1 were not. Importantly, endogenous ERKs and RSKs were also coprecipitated with FBXO15. Interaction of the kinases with UBE2R1 was similarly examined, and exogenous and endogenous RSKs were found to be coprecipitated with UBE2R1 (Fig. 2b). There was a low level of coprecipitation of exogenous ERK1 with UBE2R1, but endogenous ERKs were not detected among the immunoprecipitants. To confirm the interaction of ERK and RSK isoforms with FBXO15, HEK293 cells were cotransfected with 3 × HA-tagged *FBXO15* plasmid with each one of the *ERK1/2* or *RSK1/2/3/4* plasmids. As shown in Fig. 2c, ERK1, RSK1, RSK2 and RSK3 were coprecipitated with FBXO15. Similarly, RSK1 and RSK3 were coprecipitated with UBE2R1, but the others were not (Fig. 2d). We then examined the endogenous interaction of UBE2R1 with RSK1 (Fig. 2e). HEK293 cells were transfected with non-silencing control siRNA or si*UBE2R1*. UBE2R1 was expressed in both the cytosol and the nucleus, but RSK was expressed mostly in the cytosol. RSK1 was therefore immunoprecipitated with an isotype-specific antibody from the cytosolic fraction. Co-precipitation of UBE2R1 with RSK1 was clearly detected, and UBE2R1 disappeared in si*UBE2R1* transfectants, suggesting that RSK1 endogenously binds to UBE2R1.

### RSK1 phosphorylates Thr162 on UBE2R1

**Figure 3:**
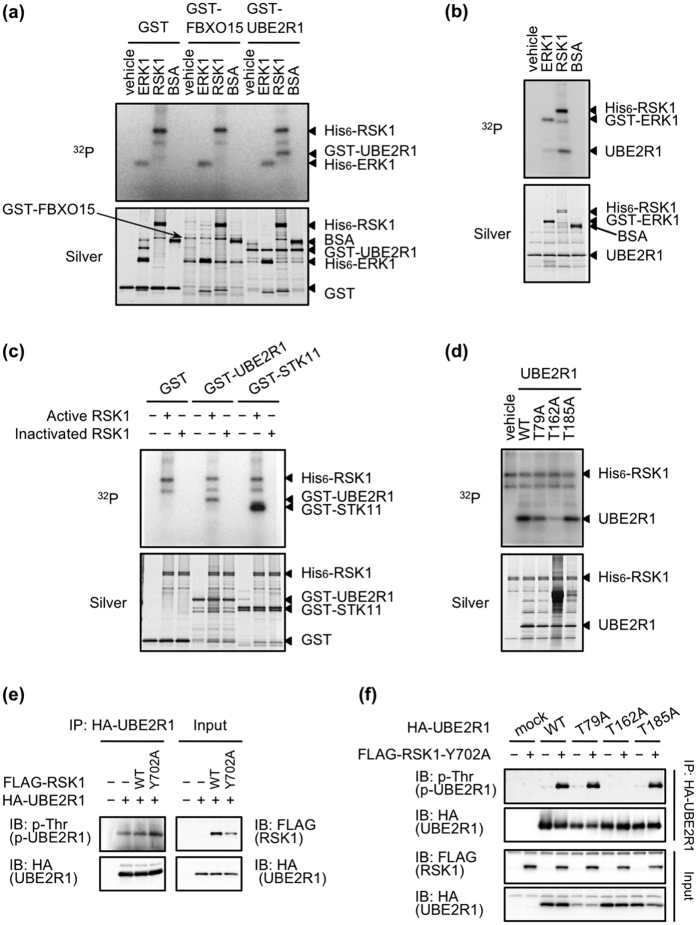
RSK1 phosphorylated Thr162 on UBE2R1. (**a–d**) An *in vitro* kinase assay using the indicated combinations of recombinant proteins. Silver staining of an SDS-PAGE gel was also performed to monitor protein loading. RSK1-mediated phosphorylation of GST-UBE2R1 (**a**); RSK1-mediated phosphorylation of tagless UBE2R1 (**b**); active RSK1-dependentt phosphorylation of GST-UBE2R1 and (**c**); searching for the phosphorylation sites of UBE2R1 by RSK1 (**d**). (**e**) RSK1-mediated phosphorylation of UBE2R1 in transfectants. HEK293 cells were transfected with the indicated combinations of plasmids for 24 h. Immunoprecipitation and immunoblotting were performed. (**f**) Confirmation of the RSK1-mediated phosphorylation site of UBE2R1 in transfectants.

An *in vitro* kinase assay was performed to examine whether ERK1 and/or RSK1 phosphorylated FBXO15 or UBE2R1. GST, GST-tagged FBXO15 and GST-tagged UBE2R1 were incubated *in vitro* in kinase reaction buffer with vehicle, active His_6_-ERK1, active His_6_-RSK1 or bovine serum albumin (BSA) in the presence of ^32^P-labeled ATP (Fig. 3a). Phosphorylated UBE2R1 was detected when it was incubated with RSK1 but not with the others. ERK1 and RSK1 did not phosphorylate FBXO15. We next confirmed the phosphorylation of tagless UBE2R1, which was generated by digestion of the GST tag after purification with glutathione sepharose, by a kinase assay *in vitro* (Fig. 3b). Again, RSK1 phosphorylated tagless UBE2R1, but ERK1 did not. To evaluate whether RSK1 kinase activity is necessary for UBE2R1 phosphorylation, active RSK1 and heat-inactivated RSK1 were used for kinase assays. GST-STK11 protein was also used as a positive control for RSK1-dependent phosphorylation^26^. As shown in Fig. 3c, active RSK1 phosphorylated both GST-UBE2R1 and GST-STK11, but heat-inactivated RSK1 did not. Next, RSK1 phosphorylation sites of UBE2R1 were assessed by kinase assays *in vitro*. As UBE2R1 possesses three phosphorylation consensus sequences of RSK, around Thr79, Thr162 and Thr185, each threonine residue was substituted with alanine (T79A, T162A or T185A, respectively). Tagless UBE2R1-WT, -T79A, -T162A and -T185A were incubated with active His_6_-RSK1 in the presence of ^32^P-labeled ATP (Fig. 3d). RSK1 phosphorylated UBE2R1-WT, -T79A and -T185A proteins but did not phosphorylate UBE2R1-T162A, suggesting that Thr162 on UBE2R1 is a phosphorylation site of RSK1. We next examined whether RSK1 phosphorylated UBE2R1 in cells. HEK293 cells were transfected with or without HA-tagged *UBE2R1*-WT plasmid combined with or without FLAG-tagged *RSK1*-WT or *RSK1*-Y702A plasmids, which has been reported as an active form^27^ (Fig. 3e). Phosphorylated UBE2R1, detected with an anti-phospho-Thr antibody, was observed in UBE2R1-expressing cells, and active mutant RSK1-Y702A clearly enhanced the phosphorylation level of UBE2R1. Similar to the *in vitro* kinase assay, RSK1-mediated phosphorylation of UBE2R1 was observed in cells expressing UBE2R1-WT, -T79A or -T185A but not in cells expressing UBE2R1-T162A (Fig. 3f). These finding suggest that RSK1 phosphorylated UBE2R1 *in vitro* and in cells, and the phosphorylation site was Thr162 on UBE2R1.

### RSK1 induced self-ubiquitination and destabilisation of UBE2R1 by phosphorylation

**Figure 4:**
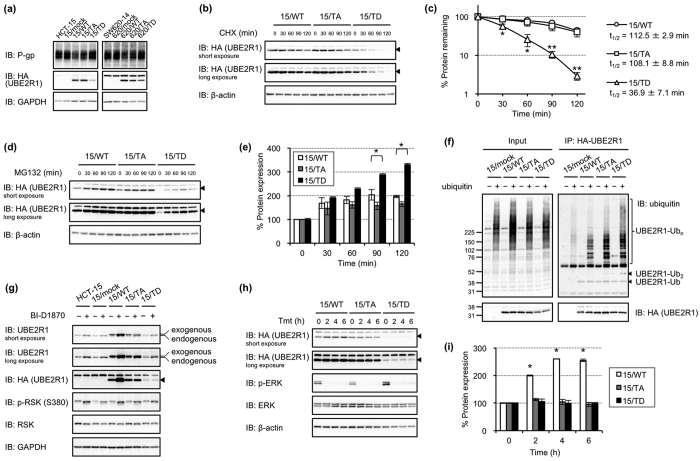
RSK1 induced self-ubiquitination and destabilisation of UBE2R1 by phosphorylation. (**a**) P-gp and UBE2R1 expression in UBE2R1-transduced cells. (**b,c**) Changes of UBE2R1 expression in cells treated with CHX for 0−120 min. Band intensities were measured using a densitometer, and each expression level relative to that of control cells (0 min) was calculated. Each point represents the mean ± SD (*N* = 3; *P < 0.05 and **P < 0.01, Student’s *t*-test, compared with 15/WT cells). (**d,e**) Changes of UBE2R1 expression in cells treated with MG132 for 0–120 min. Band intensities were measured using a densitometer, and each expression level relative to that of control cells (0 min) was calculated. Each bar represents the mean ± SD (*N* = 3; *P < 0.05, Student’s *t*-test, compared with 15/WT cells). (**f**) Changes of UBE2R1 ubiquitination. Cells were transfected with *ubiquitin* plasmid for 24 h and then treated with MG132 for 6 h. (**g**) Changes of UBE2R1 expression in cells treated with an RSK inhibitor, BI-D1870, for 6 h. (**h,i**) Changes of UBE2R1 expression in cells treated with Tmt for 0−6 h. Band intensities were measured using a densitometer, and each expression level relative to that of control cells (0 h) was calculated. Each bar represents the mean ± SD (*N* = 3; *P < 0.001, Student’s *t*-test, compared with 15/WT cells at 0 h).

To examine the role of RSK1-mediated UBE2R1 phosphorylation, we generated HCT-15 cells transduced with empty-retrovirus or UBE2R1-WT, -T162A or -T162D-expressing retroviruses, which are referred to as 15/mock, 15/WT, 15/TA or 15/TD, respectively. Similarly, 620/mock, 620/WT, 620/TA and 620/TD cells were established for SW620-14 cells. Immunoblotting with an anti-MDR1+3 antibody revealed that P-gp expression in 15/WT and 15/TA cells was lower than that in HCT-15, 15/mock and 15/TD cells (Fig. 4a, left). In addition, the expression level of UBE2R1 in 15/TD cells was lower than those in 15/WT and 15/TA cells. 620/WT and 620/TA cells also expressed lower levels of P-gp than SW620-14, 620/mock and 620/TD, and expression level of UBE2R1 in 620/TD cells was lower than in the others (Fig. 4a, right). These expressions were inversely correlated in both cell lines. Next, the stability of UBE2R1 in these cells was examined. 15/WT, 15/TA and 15/TD cells were treated with cycloheximide (CHX) and harvested every 30 min (Fig. 4b). Immunoblotting analysis revealed that UBE2R1-T162D disappeared significantly faster than UBE2R1-WT and -T162A. Each band was measured using a densitometer, and the ratio of remaining UBE2R1 proteins was plotted, as shown in Fig. 4c. The calculated half-life of UBE2R1-T162D was 36.9 ± 7.1 min, whereas those of UBE2R1-WT and -T162A were 112.5 ± 2.9 and 108.1 ± 8.8 min, respectively. By contrast, treatment with MG132 increased UBE2R1 expression, and the increasing rates of UBE2R1-T162D at 90 and 120 min were significantly higher than those of UBE2R1-WT (Fig. 4d,e). Next, self-ubiquitination of UBE2R1 was examined in cells transfected with *ubiquitin* plasmid and then treated with MG132 for 6 h (Fig. 4f). The level of poly-ubiquitinated UBE2R1, which was detected by immunoblotting with an antibody against ubiquitin after immunoprecipitation of UBE2R1, was enhanced in 15/TD cells compared with those in 15/WT and 15/TA cells. The expression level of UBE2R1 was investigated in cells treated with a RSK inhibitor BI-D1870 (Fig. 4g). BI-D1870 increased endogenous UBE2R1 expression in all cells tested, but exogenous expression of UBE2R1 increased only in 15/WT cells. Finally, the effect of trametinib on UBE2R1 expression was examined in time-course experiments (Fig. 4h,i). 15/WT, 15/TA and 15/TD cells were treated with trametinib and harvested every 2 h. Immunoblotting revealed that trametinib significantly enhanced the UBE2R1-WT expression of 15/WT cells but did not upregulate UBE2R1-T162A and -T162D proteins. These findings suggest that the MEK-ERK-RSK pathway induces self-ubiquitination of UBE2R1 and promotes its rapid degradation by phosphorylating Thr162 of UBE2R1.

### UBE2R1-T162D did not downregulate P-gp

**Figure 5:**
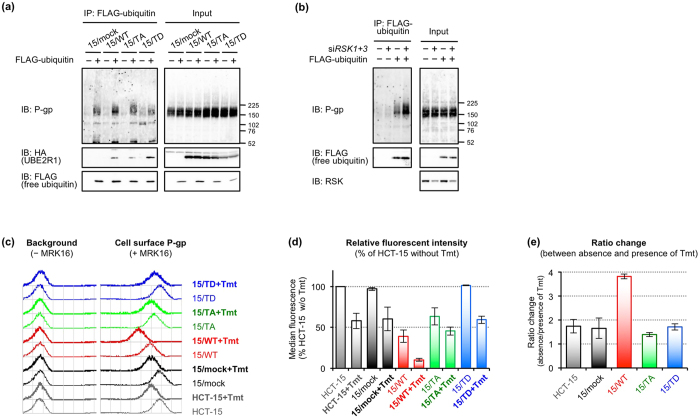
UBE2R1-T162D did not downregulate P-gp. (**a**) Changes of P-gp ubiquitination in UBE2R1-transduced cells. Cells were transfected with or without *ubiquitin* plasmid for 24 h and then treated with MG132 for 6 h. (**b**) Changes of P-gp ubiquitination in RSK1+3-knocked down cells. Cells were transfected with non-silencing control siRNA or si*RSK1+3* for 2 days and then transfected with *ubiquitin* plasmid for 24 h. Cells were treated with MG132 for 6 h just before harvest. (**c–e**) Changes of cell surface P-gp expression in UBE2R1-transduced cells. Flow cytometric analysis was performed using an anti-MDR MRK16 antibody. Median peak values were analysed using CellQuest software, and fluorescent intensities in each cell line relative to that in untreated HCT-15 cells were calculated (**d**). Relative fluorescent intensity in the absence of Tmt to that in the presence of Tmt was calculated and is presented as ratio change (**e**). Each bar represents the mean ± standard error from two independent experiments.

**Figure 6:**
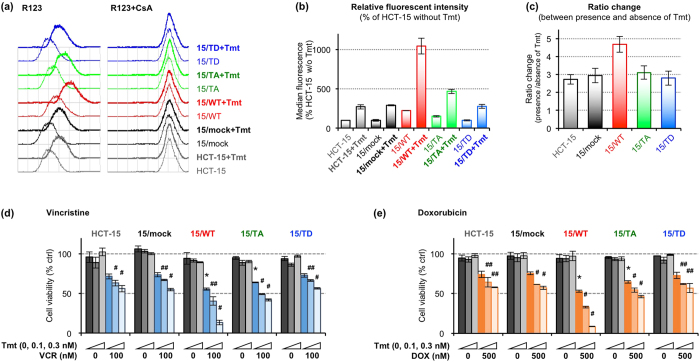
UBE2R1-T162D did not lower P-gp-mediated resistance to anticancer agents. (**a–c**) Intracellular rhodamine 123 accumulation in UBE2R1-transduced cells. Cells were treated with or without 10 nmol/L Tmt for 20 h. Trypsinised cells were incubated with 300 nmol/L rhodamine 123 combined with or without 10 μmol/L cyclosporine A (CsA) for 45 min at 37 °C with rocking and analysed using a flow cytometer (**a**). Median peak values were analysed using CellQuest software, and fluorescent intensities in each cell line relative to that in untreated HCT-15 cells were calculated (**b**). Fluorescent intensity in the presence of Tmt relative to that in the absence of Tmt was calculated and is presented as ratio change (**c**). Each bar represents the mean ± standard error from two independent experiments. (**d,e**) Sensitivity of UBE2R1-transduced HCT-15 cells to vincristine or doxorubicin in the presence or absence of trametinib. Cells were treated with 100 nmol/L vincristine (**d**) or 500 nmol/L doxorubicin (**e**) combined with or without the indicated concentrations of trametinib (0.1 or 0.3 nmol/L) for 3 days. Viable cells were measured by WST-8 assay, and the relative viabilities of cells treated with vincristine or doxorubicin relative to those of untreated cells were calculated. Each represented bar is shown as the mean ± SD (*N* = 3; *P < 0.05 compared with HCT-15 treated with vincristine or doxorubicin alone; ^#^*P* < 0.01 and ^##^*P* < 0.05 compared with cells treated with vincristine or doxorubicin alone in each cell line, Student’s *t*-test).

We examined the ubiquitination of P-gp in 15/mock, 15/WT, 15/TA and 15/TD cells. Cells were transfected with *ubiquitin* plasmid and then treated with MG132 for 6 h before harvest. Ubiquitinated P-gp was detected by immunoblotting with an anti-MDR1+3 antibody after immunoprecipitation of ubiquitin (Fig. 5a). The ubiquitinated P-gp was increased in 15/WT and 15/TA cells, but not in 15/TD cells, compared with that in 15/mock cells, suggesting that UBE2R1-T162D lacks the ability to conjugate ubiquitin to P-gp due to its self-ubiquitination and rapid degradation (Fig. 4). The effect of *RSK1* and *RSK3* knockdown on the ubiquitination of P-gp was also evaluated because they interacted with UBE2R1 (Fig. 2). Increased ubiquitination of P-gp was observed in the *RSK1+3*-knocked down cells compared with that in cells transfected with non-silencing siRNA in the presence of MG132 (Fig. 5b), whereas the ubiquitinated P-gp was not detected in the absence of MG132 (Supplementary Fig. S2). Flow cytometric analysis revealed that cell surface expression of P-gp in 15/WT and 15/TA cells was lower than that in HCT-15, 15/mock and 15/TD cells in the absence of trametinib (Fig. 5c). Cell surface expression of P-gp in 620/WT and 620/TA cells was also lower than that in SW620-14, 620/mock and 620/TD cells (Supplementary Fig. S3a). In HCT-15 and its transfectants, trametinib lowered cell surface expression of P-gp in all cell lines, but its effect on 15/WT cells was clearly greater than those on the other cell lines. Specifically, the median fluorescent intensity of 15/WT cells treated with trametinib was the lowest (Fig. 5d). The ratio change, which was calculated as the ratio of the median peak values between the absence and presence of trametinib in each cell line, was 2.2-fold higher in 15/WT cells than in HCT-15 and 15/mock cells, but the ratio changes of 15/TA and 15/TD cells were similar to those of HCT-15 and 15/mock cells (Fig. 5e). In parallel with these findings, the levels of accumulation of rhodamine 123, a fluorescent substrate of P-gp, in 15/WT and 15/TA cells were higher than those in HCT-15 and 15/mock cells (Fig. 6a). By contrast, the level of rhodamine 123 in 15/TD cells was similar to that in HCT-15 cells (Fig. 6a). Treatment with trametinib increased the levels of accumulation of rhodamine 123 in each cell line (Fig. 6a), and the effect on 15/WT cells was higher than those on the other cell lines (Fig. 6b,c). Similar results were also obtained in SW620-14 and its transfectants in the absence of trametinib (Supplementary Fig. S3b). Finally, we performed cell growth inhibition assays in the presence of vincristine or doxorubicin, which are substrate anticancer agents of P-gp, combined with trametinib. Before these experiments, we determined the concentrations of trametinib in HCT-15 and its transfectants. Cells were treated with increasing concentrations of trametinib for 3 days, and surviving cells were measured by WST-8 assay (Supplementary Fig. S4). All cell lines maintained viability of 80% or more upon treatment with trametinib at concentrations of 0.3 nmol/L. We therefore considered that 0.1 and 0.3 nmol/L trametinib did not affect cell growth and used these concentrations in subsequent experiments. HCT-15 and its transfectants were treated with 100 nmol/L vincristine or 500 nmol/L doxorubicin, both of which are the concentrations associated with approximately 20% growth inhibition in HCT-15 cells, combined with or without 0.1 or 0.3 nmol/L trametinib for 3 days. Cell viabilities were then measured by a WST-8 assay (Fig. 6d,e). Cell viabilities of 15/mock and 15/TD cells were nearly equivalent to that of HCT-15 cells upon vincristine or doxorubicin treatment. However, the cell viability of 15/WT and 15/TA cells was significantly lower than that of HCT-15 and 15/mock cells. Similar results were obtained in SW620-14 transfectants treated with 10 nmol/L vincristine or 50 nmol/L doxorubicin, both of which were IC_50_ concentrations in SW620-14 cells, in the absence of trametinib for 3 days (Supplementary Fig. S3c). Treatment with trametinib further decreased the proportion of surviving cells in a dose-dependent manner in all HCT-15 cell lines (Fig. 6d,e). The effect of trametinib on 15/WT cells was clearly greater than those on the other cell lines. These findings indicate that UBE2R1-WT or -T162A-expressing cells acquired additional ubiquitin-conjugating activity for P-gp and downregulated its expression along with increasing sensitivity to vincristine and doxorubicin, but UBE2R1-T162D-expressing cells did not. In addition, trametinib lowered P-gp-mediated resistance to anticancer agents by upregulating endogenous UBE2R1 in all cell lines, and it additionally decreased the resistance by exogenous UBE2R1 in UBE2R1-WT-expressing cells alone.

## Discussion

**Figure 7:**
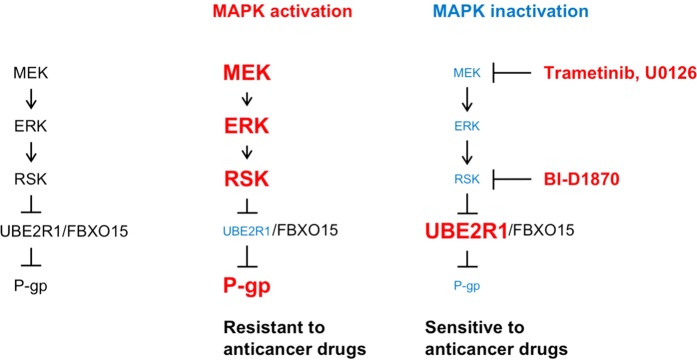
Schematic of the MAPK-mediated P-gp regulation through UBE2R1 expression. Activation of MAPK signalling promotes the degradation of UBE2R1 by its self-ubiquitination, resulting in the upregulation of P-gp. By contrast, inactivation of MAPK signalling by small-molecule inhibitors, such as trametinib, U0126 and BI-D1870, upregulates UBE2R1 expression and downregulates P-gp expression.

We previously reported that MEK inhibition by U0126 or knockdown of *MEK1/2*, *ERK1/2* or *RSK1/2/3* lowers P-gp expression without affecting *MDR1* mRNA expression^21^. Pulse-chase labelling with [^35^S]methionine/cysteine revealed that U0126-mediated downregulation of P-gp was caused by the promotion of its rapid disappearance^21^. We also investigated the degradation system of P-gp and found two mechanisms: (1) cell surface P-gp disappears by lysosomal and proteasomal degradation^28^ after endocytosis-dependent intracellular trafficking; and (2) P-gp is degraded by the ubiquitin–proteasome system, which is mediated by UBE2R1-SCF^Fbx15 22^. In this study, crosstalk between the MAPK inhibition-mediated P-gp degradation and the ubiquitin–proteasome system was investigated, and we successfully demonstrated that RSK1 induces self-ubiquitination of UBE2R1 and its rapid degradation in a phosphorylation-dependent manner, resulting in protection against the ubiquitin–proteasomal degradation of P-gp (Figs 3, 4, 5). In particular, RSK1 phosphorylated UBE2R1-WT but not UBE2R1-T162A (Fig. 3); the phosphorylation-mimicking mutant UBE2R1-T162D rapidly disappeared (Fig. 4b,c) by increasing self-ubiquitination (Fig. 4g); MEK inhibitors, trametinib and U0126 (Figs 1b and 4h, and Supplementary Fig. S1b), and an RSK inhibitor, BI-D1870 (Fig. 4g), upregulated UBE2R1-WT but not its mutants; and knockdown of RSK1 and RSK3 increased P-gp ubiquitination (Fig. 5b). In addition, UBE2R1-T162D expression did not downregulate P-gp (Figs 4a and 5c, and Supplementary Fig. S3a). These results suggest that RSKs protect against P-gp ubiquitination and degradation by modulating UBE2R1 expression. Hence, MAPK signalling is one of the most important pathways in regulating P-gp expression. Activation of MAPK signalling, which is often observed in cancer cells, promotes the degradation of UBE2R1 by its self-ubiquitination, resulting in the upregulation of P-gp. By contrast, inactivation of MAPK signalling by small-molecule inhibitors, such as trametinib, U0126 and BI-D1870, upregulates UBE2R1 expression and downregulates P-gp expression (Fig. 7).

As another type of post-translational regulation of P-gp, we also reported that the complex of protein phosphatase 5 (PP5) and protein phosphatase 2A, regulatory subunit B, gamma (PPP2R3C) downregulates P-gp expression^29^. PKA isoform, type II (PKA-RIIα) and A-kinase anchoring protein (AKAP350) modulate P-gp trafficking to the apical canalicular membrane, and PKC regulates cell volume-activated chlorine channels through P-gp phosphorylation^30^,^31^,^32^. These kinases phosphorylate a couple of serine residues in the intracellular linker region of P-gp, and the PP5-PPP2R3C complex negatively regulates P-gp expression by dephosphorylating these sites^29^. In parallel with the current study, we examined the crosstalk between PP5-PPP2R3C and kinases involved in the MAPK signalling. However, no such kinases co-precipitated with PP5 or PPP2R3C (data not shown). Therefore, the downregulation of P-gp by the PP5-PPP2R3C complex is thought to be independent of that by MEK inhibitors.

UBE2R1 also ubiquitinates WEE1 and p27^KIP1^ and promotes their proteasomal degradation^33^,^34^. WEE1 and p27^KIP1^ are negative regulators of cell cycle progression at the G2/M transition and at both G1 and G2/M phases, respectively^17^,^35^,^36^. UBE2R1 is therefore involved in cell cycle progression by the elimination of WEE1 and p27^KIP1^. BI-D1870 has been reported to arrest HCT 116 and oral squamous cell carcinoma cell lines at G2/M^37^,^38^. As our data show that BI-D1870 increases the stability of UBE2R1 (Fig. 4h), BI-D1870 might affect cell cycle progression at the G2/M transition by controlling the expression and activity of UBE2R1.

P-gp is expressed in normal cells and tissues, including colon, kidney, liver, small intestine, and the blood–brain and blood–placenta barriers^10^,^12^. P-gp protects them against many different physiologically active or toxic substances. Trametinib (Mekinist^®^, developed by Japan Tobacco, Inc. and GlaxoSmithKline) and bortezomib (Velcade^®^, developed by Millennium Pharmaceuticals, Inc.) have already been approved and clinically used for melanomas harbouring BRAF V600 mutations and refractory multiple myelomas, respectively^39^,^40^,^41^,^42^,^43^. As our data show that trametinib and bortezomib modulate P-gp expression (Fig. 1), they might cause the changes in pharmacokinetics and pharmacodynamics of the drugs that are substrates of P-gp. Patients with progressive melanomas receive treatment with trametinib combined with dacarbazine or paclitaxel^44^. In these cases, the pharmacokinetics of paclitaxel might fluctuate because it is one of the substrates of P-gp.

More importantly, our data show that trametinib downregulates the expression of P-gp by promoting its degradation through the upregulation of UBE2R1, resulting in the downregulation of P-gp-mediated resistance to the anticancer agents vincristine and doxorubicin (Fig. 6d,e). Trametinib would thus be promising for overcoming chemoresistance mediated by P-gp in clinical cancers. In addition, BI-D1870 shows higher upregulation of UBE2R1 than trametinib. A potential reason for this is that RSK is a direct regulator of UBE2R1 (Figs 2 and 3), and there might be some mechanism(s) by which the regulation of UBE2R1 can be bypassed between MEK and RSK. RSK inhibitors would be more powerful modulators of P-gp expression via UBE2R1 than trametinib, and they would have great potential for development for use in a clinical context.

Overall, this study should be beneficial not only for understanding the molecular mechanism of P-gp expression but also for predicting the side effects of drugs used in combination with trametinib or bortezomib and for overcoming P-gp-mediated chemoresistance to anticancer agents.

## Methods

### Reagents and recombinant proteins

MG132, CHX, doxorubicin, cyclosporine A and recombinant GST-ERK1 were purchased from Sigma-Aldrich (St. Louis, MO, USA). U0126 and bortezomib were obtained from Cell Signaling Technologies (Danvers, MA, USA) and Takeda Pharmaceutical Company Ltd. (Osaka, Japan), respectively. Trametinib and BI-D1870 were from Selleck Chemicals (Houston, TX, USA). Recombinant His_6_-ERK1 and His_6_-RSK1 were purchased from Merck Millipore (Billerica, MA, USA).

### siRNAs, plasmids and transfection

Non-silencing control siRNA was purchased from Qiagen (Hilden, Germany), and *FBXO15*- or *CDC34/UBE2R1*-targetting siRNAs were from GE Healthcare UK Ltd. (Little Chalfont Bucks, UK).

pQCXIP-UBE2R1, pGEX-UBE2R1 and pGEX-FBXO15 were generated by the digestion of pcDNA3.1-HA-UBE2R1 or -FBXO15, which had been generated previously^22^, with *Xba*I and *Not*I, followed by ligation of each cDNA to pQCXIP or pGEX-6P-3 vectors. Substitution of each amino acid in UBE2R1 was performed using QuikChange II Site-Directed Mutagenesis Kit (Agilent Technologies, Santa Clara, CA, USA), in accordance with the manufacturer’s instructions. All other plasmids had also been generated previously^21^.

FuGENE HD transfection reagent (Promega, Madison, WI, USA) and Lipofectamine 2000 transfection reagent (Invitrogen, Carlsbad, CA, USA) were used for the transfection of cells with plasmids and siRNAs, respectively, in accordance with the manufacturers’ instructions.

### Cells

SW620-14 cells had been previously isolated from human colorectal cancer SW620 cells^21^. 15/mock, 15/WT, 15/TA and 15/TD cells were established from human colorectal cancer HCT-15 cells by transduction with the QCXIP retroviruses alone or coding *UBE2R1*-WT, -T162A or -T162D, respectively. Similarly, 620/mock, 620/WT, 620/TA and 620/TD cells were established based on SW620-14 cells. All cell lines including human embryonic kidney HEK293 cells were maintained in DMEM supplemented with 7% FBS and 50 μg/mL kanamycin at 37 °C in 5% CO_2_.

### Flow cytometric analysis and cell growth inhibition assay

Flow cyctometric analyses and cell growth inhibition assays were performed as described previously^21^,^22^,^29^. Flow cytometric results were subject to statistical analyses using CellQuest software (Beckon-Dickinson Biosciences, San Jose, CA, USA).

### Immunoprecipitation and immunoblotting

Immunoprecipitation and immunoblotting were performed as described previously^21^,^22^,^29^. Anti-FLAG M2 or anti-HA affinity gels (Sigma-Aldrich) were used for immunoprecipitation of FLAG- or HA-tagged proteins by the incubation of cell lysates with these affinity gels for 2 h or overnight at 4 °C with rocking. Immunoprecipitants were eluted by incubation with the corresponding peptides (Sigma-Aldrich) for 30 min at room temperature with vortexing. For immunoprecipitation of RSK1, precleared cell lysates were incubated with anti-RSK1 antibody (Santa Cruz Biotechnology, Santa Cruz, CA, USA) for 2 h at 4 °C, followed by GammaBind G Sepharose (GE Healthcare UK Ltd.) overnight at 4 °C with rocking. The following antibodies were used for immunoblotting: an anti-MDR1+3 antibody (C219; Abcam, Cambridge, UK); anti-FBXO15, anti-β-actin and peroxidase-conjugated anti-FLAG M2 antibodies (Sigma-Aldrich); anti-MEK, anti-ERK, anti-phospho-ERK (T202/Y204), anti-RSK, anti-phospho-RSK (S380), anti-UBC3 and anti-phospho-threonine (Thr) antibodies (Cell Signaling Technology); a peroxidase-conjugated anti-HA antibody (3F10; Roche Applied Science, Penzberg, Germany); an anti-GAPDH antibody (6C5; Merck Millipore); and an anti-ubiquitin antibody (1B3; Medical and Biological Laboratories, Nagoya, Japan). The band intensities of UBE2R1 were quantified using the ImageJ densitometric program (NIH, Bethesda, MD, USA) and normalised with those of GAPDH. Each point or column represents the mean ± standard deviation (SD) from three independent experiments.

### *In vitro* kinase assay

GST-tagged UBE2R1 or FBXO15 purified from *E. coli* with Glutathione Sepharose 4B (GE Healthcare UK Ltd.) was incubated with BSA or GST-ERK1, His_6_-ERK1 or His_6_-RSK1 in kinase reaction buffer [20 mmol/L MOPS (pH 7.0), 25 mmol/L β-glycerophosphate, 5 mmol/L EGTA, 1 mmol/L sodium orthovanadate, 1 mmol/L DTT, 112.5 μmol/L ATP and 17 mmol/L MgCl_2_] for 30 min at 30 °C in the presence of 40 μCi [γ-^32^P]ATP. The reaction was stopped by incubation for 3 min on ice followed by the addition of 5 × Laemmli sample buffer and boiling for 3 min at 95 °C. The proteins were subjected to SDS-PAGE, and the incorporated radioactivity was visualised by autoradiography using an image reader FLA-7000 (GE Healthcare UK Ltd.). To confirm that the proteins were present at equal levels, the gel was subjected to silver staining.

## Additional Information

**How to cite this article**: Katayama, K. *et al*. RSK1 protects P-glycoprotein/ABCB1 against ubiquitin-proteasomal degradation by downregulating the ubiquitin-conjugating enzyme E2 R1. *Sci. Rep*. **6**, 36134; doi: 10.1038/srep36134 (2016).

**Publisher’s note:** Springer Nature remains neutral with regard to jurisdictional claims in published maps and institutional affiliations.

## Supplementary Material

Supplementary Information
