# Supplementary material for: RSK1 protects P-glycoprotein/ABCB1 against ubiquitin–proteasomal degradation by downregulating the ubiquitin-conjugating enzyme E2 R1
Source: Sci Rep. 2016 Oct 27;6:36134. doi: 10.1038/srep36134 (PMC5081560; doi:10.1038/srep36134)
Supplement: Supplementary Information [file srep36134-s1.pdf]

RSK1 protects P-glycoprotein/ABCB1 against ubiquitin–proteasomal degradation by downregulating the ubiquitin-conjugating enzyme E2 R1

Running title: RSK1 downregulates UBE2R1 and prevents P-gp degradation

Kazuhiro Katayama, Chiaki Fujiwara, Kohji Noguchi, Yoshikazu Sugimoto*

Division of Chemotherapy, Faculty of Pharmacy, Keio University, Tokyo, Japan.

*Corresponding author:

Yoshikazu Sugimoto, Division of Chemotherapy, Faculty of Pharmacy, Keio University, 1-5-30 Shibakoen, Minato-ku, Tokyo 105-8512, Japan.

E-mail: sugimoto-ys@pha.keio.ac.jp

Tel & Fax: +81-3-5400-2669

Number of supplementary figures: 4

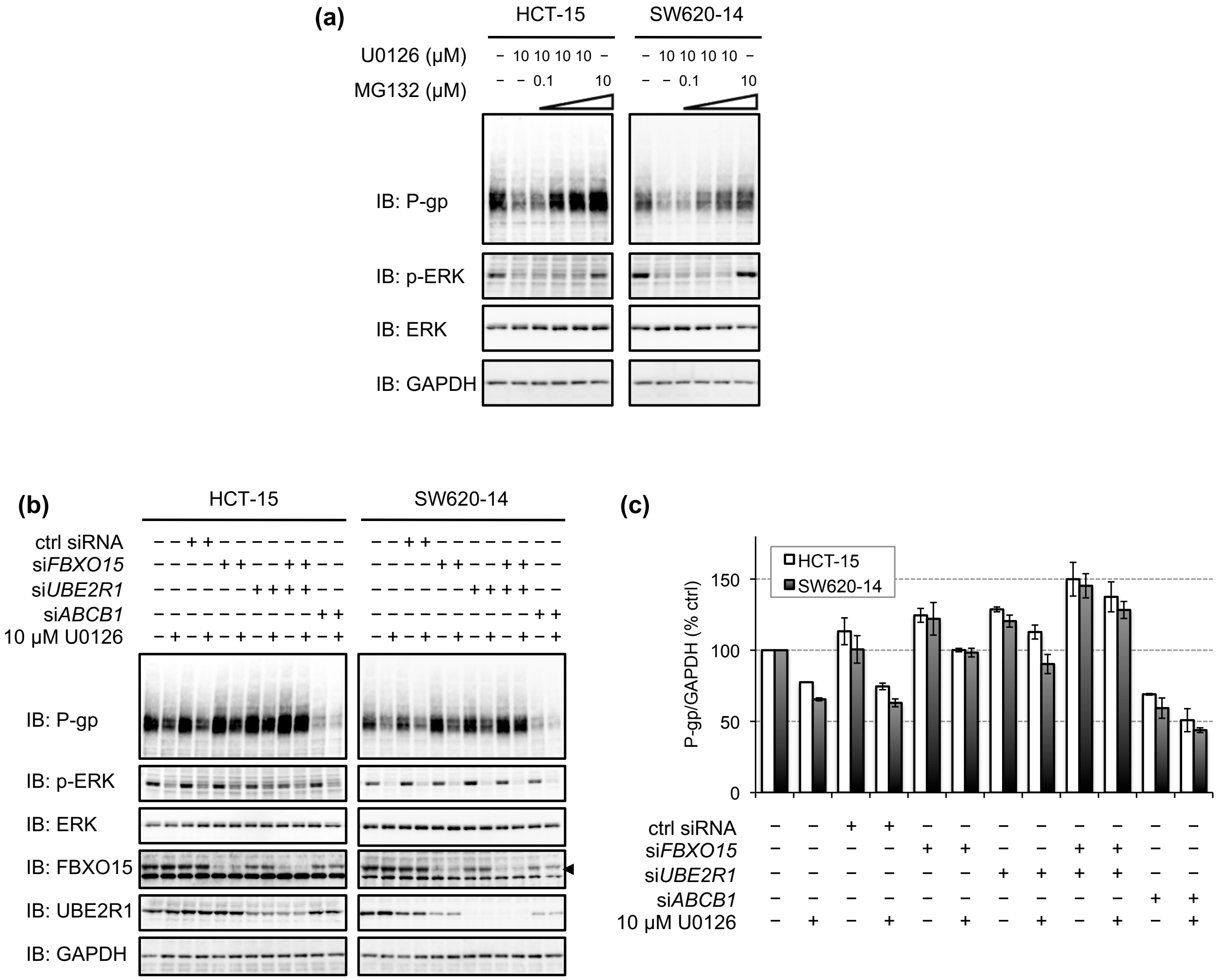

Supplementary Figure S1: MG132 or FBXO15/UBE2R1 knockdown reduced U0126-mediated downregulation of P-gp. (a) Changes of P-gp expressions in cells treated with U0126 and/or MG132 for 10 h. **(b)** Changes of P-gp expression in cells transfected with si*FBXO15* and/or si*UBE2R1* for 60 h followed by treatment with U0126 for 10 h. **(c)** Graphic representation of P-gp expression normalized by GAPDH expression in (b). Each represented bar is shown as the mean ± error from two independent experiments.

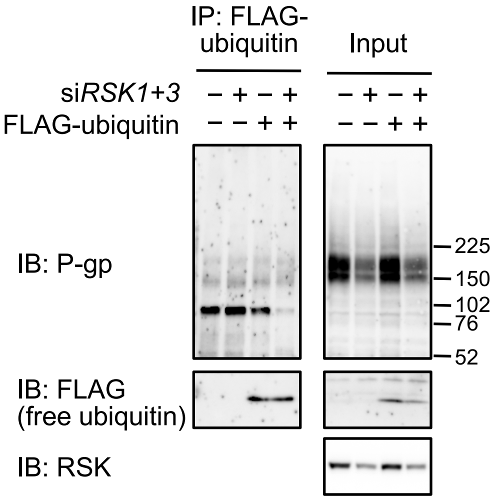

Supplementary Figure S2: Ubiquitination assay of P-gp in the RSK1+3-knocked down cells in the absence of proteasome inhibitors. Cells were transfected with non-silencing control siRNA or si*RSK1+3* for 2 days and then transfected with *ubiquitin* plasmid for 24 h. Cells treated without any proteasome inhibitors were harvested, and FLAG-ubiquitin was immunoprecipitated with an anti-FLAG M2 antibody. P-gp was detected by western blotting using an anti-MDR C219 antibody. An asterisk shows background bands derived from immunoglobulin used in immunoprecipitation.

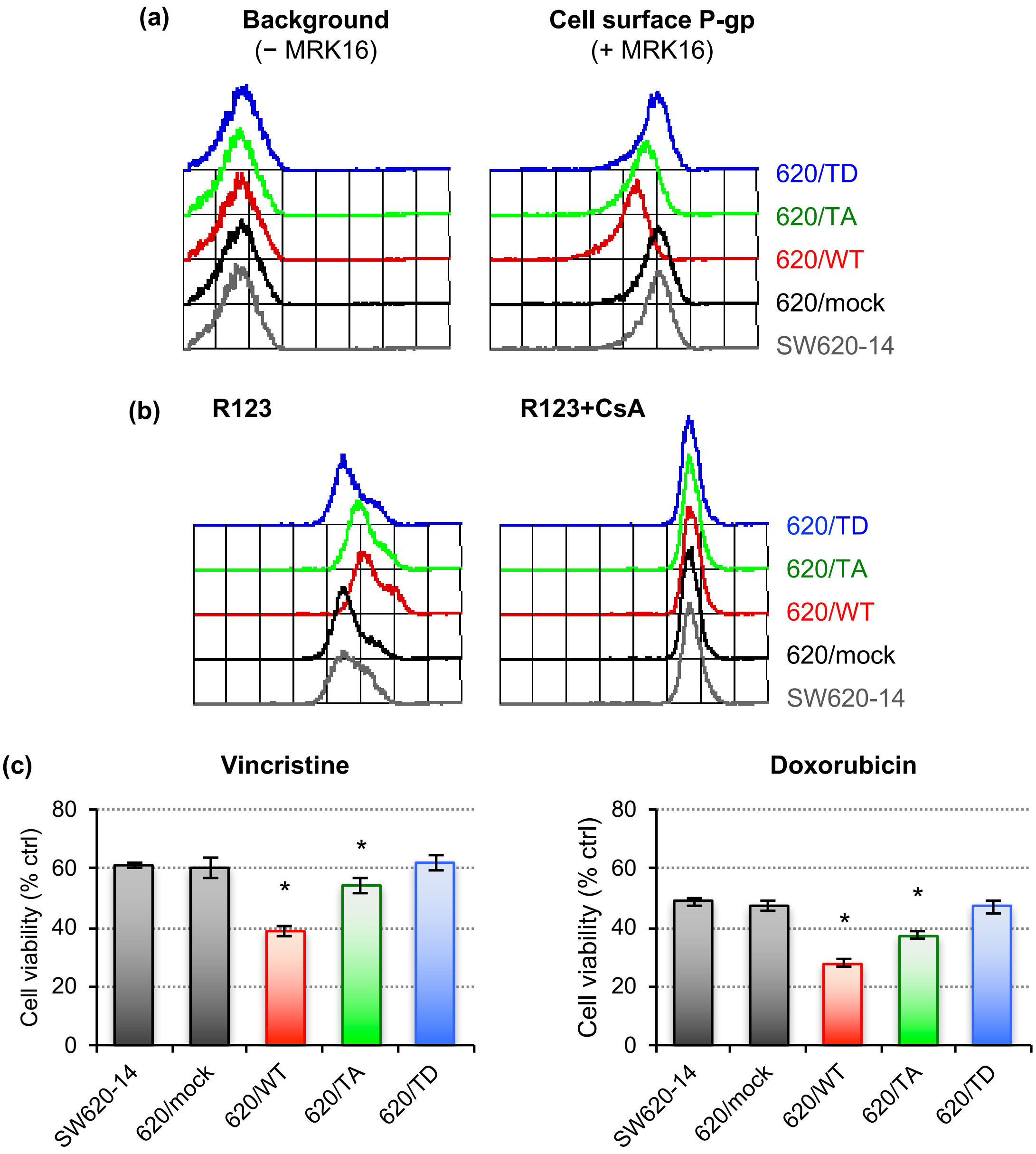

Supplementary Figure S3: Cell surface P-gp expression, rhodamine 123 accumulation and growth inhibition assay in the UBE2R1-transduced SW620-14 cells. (a) Changes of cell surface P-gp expression in UBE2R1-transduced SW620-14 cells. Flow cytometric analysis was performed using an anti-MDR MRK16 antibody. **(b)** Intracellular rhodamine 123 accumulation in UBE2R1-transduced SW620-14 cells. Trypsinised cells were incubated with 300 nmol/L rhodamine 123 combined with or without 10 μmol/L cyclosporine A (CsA) for 45 min at 37°C with rocking and analysed using a flow cytometer. **(c)** Sensitivity of UBE2R1-transduced SW620-14 cells to vincristine or doxorubicin in the presence or absence of trametinib. Cells were treated with 10 nmol/L vincristine or 50 nmol/L doxorubicin for 3 days. Viable cells were measured by WST-8 assay, and the viabilities of cells treated with vincristine or doxorubicin relative to those of untreated cells were calculated. Each represented bar is shown as the mean ± SD (N=6; *P<0.001, Student's t-test).

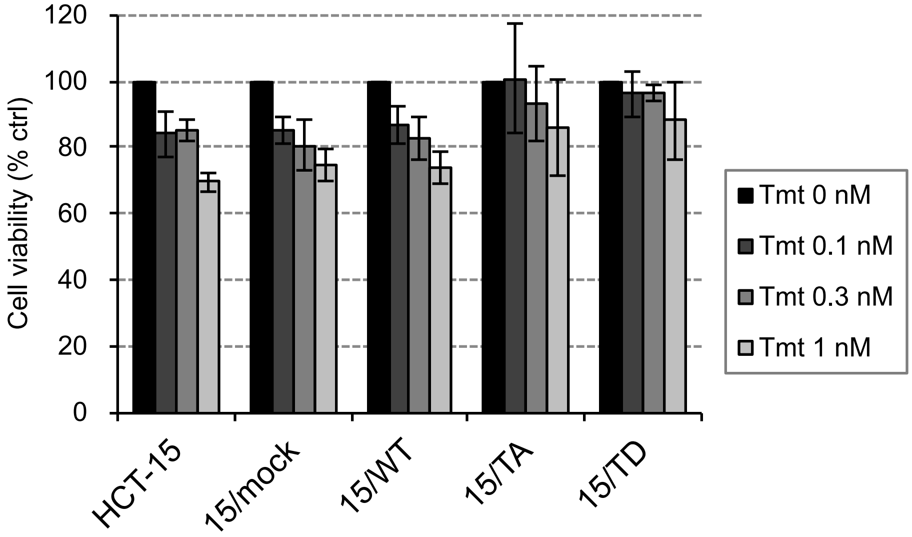

Supplementary Figure S4: Viability of cells treated with or without trametinib. Cells were treated with or without 0.1−1 nM trametinib for 3 days, and WST-8 assay was performed to determine viable cells. Treatment with trametinib (≤ 0.3 nM) allowed 80% or more viability in all cell lines tested.
